# Atorvastatin Solid Lipid Nanoparticles as a Promising Approach for Dermal Delivery and an Anti-inflammatory Agent

**DOI:** 10.1208/s12249-020-01807-9

**Published:** 2020-09-25

**Authors:** Seyed Sadegh Shahraeini, Jafar Akbari, Majid Saeedi, Katayoun Morteza-Semnani, Shidrokh Abootorabi, Milad Dehghanpoor, Seyyed Sohrab Rostamkalaei, Ali Nokhodchi

**Affiliations:** 1 Department of Pharmaceutics, Faculty of Pharmacy, Mazandaran University of Medical Sciences, Sari, Iran; 2 Department of Medicinal Chemistry, Faculty of Pharmacy, Mazandaran University of Medical Sciences, Sari, Iran; 3 Department of Pharmaceutics, Faculty of Pharmacy, Islamic Azad University, Ayatollah Amoli Branch, Amol, Iran; 4 Medicinal Plant Research Center, Faculty of Pharmacy, Islamic Azad University, Ayatollah Amoli Branch, Amol, Iran; 5 Pharmaceutics Research Laboratory, School of Life Sciences, University of Sussex, Brighton, BN1 9QJ UK

**Keywords:** solid lipid nanoparticles, atorvastatin, HLB, anti-inflammatory effects, scalp seborrheic dermatitis, inflammatory, dermal drug delivery

## Abstract

In the current research, the main focus was to overcome dermal delivery problems of atorvastatin. To this end, atorvastatin solid lipid nanoparticles (ATR-SLNs) were prepared by ultra-sonication technique. The prepared SLNs had a PDI value of ≤ 0.5, and the particle size of nanoparticles was in the range 71.07 ± 1.72 to 202.07 ± 8.40 nm. It was noticed that, when the concentration of lipid in ATR-SLNs increased, the size of nanoparticles and drug entrapment efficiency were also increased. Results showed that a reduction in the HLB of surfactants used in the preparation of SLN caused an increase in the particle size, zeta potential (better stability), and drug entrapment efficiency. Despite Tween and Span are non-ionic surfactants, SLNs containing these surfactants showed a negative zeta potential, and the absolute zeta potential increased when the concentration of Span 80 was at maximum. DSC thermograms, FTIR spectra, and x-ray diffraction (PXRD) pattern showed good incorporation of ATR in the nanoparticles without any chemical interaction*. In vitro* skin permeation results showed that SLN containing atorvastatin was capable of enhancing the dermal delivery of atorvastatin where a higher concentration of atorvastatin can be detected in skin layers. This is a hopeful promise which could be developed for clinical studies of the dermal delivery of atorvastatin nanoparticles as an anti-inflammatory agent.

## INTRODUCTION

To achieve a better transdermal delivery, it is important to overcome skin barriers, particularly the stratum corneum (1). It has been shown that formulations containing nanoparticles can pass through the skin layers efficiently to improve the percutaneous absorption of drugs without making any modifications in the chemical assembly of drugs (2,3). Although there are many different types of nanocarriers, solid lipid nanoparticles (SLNs) have been widely used recently which is due to many advantages of SLNs over conventional formulations such as excellent physical stability, good tolerability, and ease of scale-up and manufacturing (4). As SLNs do not show any significant interaction with the stratum corneum and skin layers, and they have a strong potential to enhance the skin permeation of drugs, therefore, SLNs have been considered as a good platform for effective skin delivery for drugs (5). They are composed of lipids which are uniformly dispersed in an aqueous surfactant solution (6). Statins have anti-inflammatory effects shown in both *in vitro* and *in vivo* and evidence has shown that statins can be used for the treatment of dermatological disorders and their anti-inflammatory activity is significant in comparison with diclofenac (7,8). Atorvastatin is an anti-hyperlipidemic drug which shows a poor oral bioavailability around 12% due to first-pass metabolism (9). Atorvastatin is a highly lipophilic drug in nature (10) and its anti-inflammatory effect on topical administration is comparable with the topical formulation of betamethasone which could be an alternative drug in the treatment of scalp seborrheic dermatitis (11). Many researchers have tried to apply atorvastatin for transdermal administration (12,13) and they have used lipid nanoparticles for avoiding the first-pass metabolism of atorvastatin (9). Although many publications have shown that the type and the amount of surfactant and lipid can have an influence on SLN properties, there are only two publications that studied the effect of HLB and the ratio of Span:Tween to optimize the properties of SLN in terms of size, polydispersity index, zeta potential, and entrapment efficiency percentage (14,15). The optimized formulation is selected for further studies (permeation studies). There are no examples of using solid lipid nanoparticles to localize a highly lipophilic drug, atorvastatin, in the skin as an anti-inflammatory agent. Furthermore, this study showed how the behavior of ATR-SLN could be modulated by the HLB of the surfactants to achieve the best formulations for the dermal delivery of atorvastatin.

## MATERIALS AND METHODS

### Materials

Atorvastatin calcium (ATR) was obtained from Hakim Pharmaceuticals (Tehran, Iran). Tween 40, Span 80 and glyceryl monostearate (GMS) were obtained from Merck, Germany.

### Preparation of ATR-SLN

Atorvastatin SLNs were prepared by using the probe ultra-sonication technique. Glyceryl monostearate was melted at below 80°C and atorvastatin was mixed with molten GMS using a heater stirrer (Hei-Standard). Tween and Span were homogenized in water using a silent crusher M (Heidolph, Germany) at 7000 rpm and then heated to 80°C. The obtained binary mixture of the surfactant solution was blended dropwise with the lipid phase using a heater stirrer to form a pre-emulsion. The resultant pre-emulsion was then subjected to sonication (amplitude 20%) for 10 min using a probe sonicator (Bandelin sonoplus HD 3100, Germany) to form nano-emulsion. At the end of the sonication process, an ice bath was instantly used to turn nano-emulsion to nano-suspension and finished the process (16,17). The amounts of excipients used to make various ATR-SLNs are listed in Table I.

**Table I:** Composition and Properties of Various Atorvastatin Solid Lipid Nanoparticles (ATR-SLN) (*n* = 3, Mean ± SD)

	ATR* (%*w*/w)	GMS** (%w/w)	Tween 40 (%w/w)	Span 80 (%w/w)	Water (%w/w)	HLB	Particle Size	PDI***	ZP**** (mv)	EE (%)*****
ATR-SLN1	0.12	0.31	1.33	0.67	97.56	11.8	93.73 ± 5.65	0.48 ± 0.01	−20.46 ± 1.01	38.72 ± 1.14
ATR-SLN2	0.12	0.62	1.33	0.67	97.25	11.8	94.47 ± 3.71	0.48 ± 0.02	−16.30 ± 1.01	45.33 ± 1.19
ATR-SLN3	0.12	1.25	1.33	0.67	96.63	11.8	143.00 ± 2.19	0.43 ± 0.02	−14.20 ± 0.26	49.98 ± 0.90
ATR-SLN4	0.12	1.25	2.00	0.00	96.62	15.6	71.07 ± 1.72	0.42 ± 0.03	−7.70 ± 0.61	34.90 ± 1.30
ATR-SLN5	0.12	1.25	1.78	0.21	96.62	14.3	83.33 ± 4.67	0.43 ± 0.02	−10.23 ± 0.66	44.98 ± 1.67
ATR-SLN6	0.12	1.25	1.50	0.50	96.62	12.7	115.73 ± 1.97	0.46 ± 0.01	−12.03 ± 0.15	47.42 ± 1.44
ATR-SLN7	0.12	1.25	1.17	0.83	96.62	10.8	148.77 ± 1.55	0.43 ± 0.02	−14.73 ± 0.70	53.86 ± 1.51
ATR-SLN8	0.12	1.25	0.85	1.15	96.62	9.1	202.07 ± 8.40	0.43 ± 0.01	−17.00 ± 0.87	42.12 ± 1.39

*Atorvastatin

**Glyceryl monostearate

***Polydispersity index

****Zeta potential

*****Entrapment efficiency

### Measurement of Particle Size and Zeta Potential

Zetasizer Nano ZS (Malvern Panalytical, UK) at 25°C and a 90° detector angle were used to characterize nanoparticles with a concentration of 20–400 kilo counts per second (KCPS). The intensity of diffraction was 100,000 counts per second (18). In this technique, z-average was employed to measure the size of particles and cumulant was used for autocorrelation. In addition, the zeta potential of particles which is the sum of the initial surface charge and is established on the surface of any material when it comes in contact with a liquid medium was measured.

### Entrapment Efficiency

In this experiment in order to make sure ATR trapped in the micelles are not considered as entrapment drugs in the entrapment efficiency calculation, the drug solution containing surfactants was centrifuged for 2 h at 54950 g (Hermle, Z36HK, Germany). The surfactants were able to form micelles and dissolve the drug completely. When the supernatant was injected to HPLC to quantify the amount of ATR, the results showed that all the drug added to the solution was detectable. This indicates all the micelles stay in the supernatant layer and it is considered as a free drug (unloaded drug). In order to calculate EE%, the nano-dispersions formulations were centrifuged (2 h at 54950 g). After 2-h centrifugation, the free drug and drugs entrapped in the micelles should stay in the supernatant layer and the total of the drug in the supernatant can be determined using an HPLC method described below. The supernatant layer was filtered (pore size of 0.22 μm), followed by ATR determination in the filtered solution (free drug) by Knauer HPLC at 228 nm. In the HPLC system, acetonitrile and phosphate buffer (KH_2_PO_4_) 0.02 M at pH = 4.5 (65:35 v/v) were used as a mobile phase which was delivered at 1 ml/min. The column used in the HPLC system was Kneuer XDB-C18 (5 μm, 4.6 × 250 mm) (19) and was kept at 25°C. The volume of injection was 20 μl. Drug entrapment efficiency (EE %) was calculated using Eq. (1):

1 $$ \mathrm{EE}\%=\left[\ \left({W}_{\mathrm{id}}-{W}_{\mathrm{fd}}\ \right)/{W}_{\mathrm{id}}\right]\times 100 $$

where *W*_id_ and *W*_fd_ were the initial drug and free drug concentration respectively (20).

### Fourier Transform Infrared Spectroscopy

To identify any changes or interaction between various chemicals used in the preparation of ATR-SLNs, an FTIR spectrophotometer (PerkinElmer, USA) in the frequency range from − 4000–400 cm^−1^ at a resolution of 4 cm^−1^ was used. To make a disk with a suitable size (13 mm), KBr was used and the mixture was pressed hydraulically into transparent discs with 10 bar of pressure (18).

### Differential Scanning Calorimetry

DSC traces of samples (drug, lipid, and lyophilized ATRS-SLN) were recorded using DSC Pyris 6 (PerkinElmer, USA). The sample size was around 5 mg for each measurement and all the samples were evaluated in crimped aluminum pans. The aluminum pans containing excipients or formulations were heated from 30 to 300°C at a scanning rate of 10°C/min. Indium was used to calibrate the DSC.

### Powder X-ray Diffractometer Analysis

To investigate any changes in the solid state of the chemicals such as changes from the crystalline state to the amorphous state, PXRD (Bruker D8, Germany) was used at 40 kV and 30 mA. In this experiment, ATR, GMS, and lyophilized SLN were exposed to Cu Kα radiation of 1.5406 Å. The samples were scanned at a 2θ of 5 to 50°, and the step size was 0.040° with a step time of 1 s.

### *In Vitro* Skin Permeation Study

Wistar male rats were used in the skin permeation study (the abdominal skin of the animal was used). To prepare the abdominal rat skin, rats weighing 200–250 g were exterminated by chloroform and the abdominal skin was shaved by a scissor (the protocol was approved by the ethical committee of Mazandaran University of Medical Sciences, and the ethical code was ir.mazums.rec.94.1641). The skin was surgically excised and was placed in a beaker (for 24 h) containing a saline solution before starting the *in vitro* skin permeation study (21). The skin was set on Franz diffusion cells so that the dermis is facing the receptor. A solution of 70:30 ethanol/water mixtures was selected as a receptor compartment. The temperature of diffusion cells was set at 32 ± 0.5°C (skin temperature) with a circulator bath and the stirring set at 150 rpm. In this study, ATR-SLNs were compared with atorvastatin solution where ethanol 70% was used as a solvent. To calculate the amount of ATR permeated through the skin within 24 h, a 5-ml solution from the receptor compartment was withdrawn and an equivalent volume of the fresh medium maintained at 32°C was added to the receptor compartment to keep the final volume of the receptor compartment constant. To calculate the amount of ATR penetrating and targeting the skin layers (when the *in vitro* permeation test was finished), the used skins in the diffusion study were collected from Franz diffusion cells and the surface of the skin was rinsed three times with purified water. The skin was minced and immersed in a 70:30 ethanol/water mixture for 24 h and then sonicated for three times (each time for 22 min) with a bath sonicator to remove ATR deposited within the skin. The clear solution in the samples was then separated and filtered through a 0.22-μm syringe filter and the amount of drug determined by HPLC at 228 nm which was described earlier in this article.

### Scanning Electron Microscopy

SNE-4500M bench-top scanning electron microscope (Scitek, Australia) was used to study the morphology of nanoparticles which was metalized with 10 nm of platinum. The observation was performed at 20 kV.

### Atomic Force Microscopy

JPK-AFM (JPK Instruments AG, Berlin, Germany) was used to analyze the shape and morphology of nanoparticles. Nanoparticles were diluted 500 times and dropped on the lame and dried at room temperature. Contact mode was selected, and photos were made by repulsive forces.

### Statistical Analysis

The results presented in the table are the means and standard deviation of three determinations (*n* = 3). To investigate whether a specific parameter has any effect on the properties of ATR-SLN formulations, non-parametric analysis (Kruskal-Wallis test with a post-test) was used. In case of any significant results, a multiple comparison test (Tukey test) was also employed to show a significant difference between two formulations (the treated groups with the control). *T* test was also performed to compare a significant difference between two skin penetrations and permeation after 24 h. SPSS software 20 was used for all the statistical analyses.

## RESULTS AND DISCUSSION

### Effect of Lipid Concentration on Nanoparticle Characterization

Table I shows the composition and properties of the ATR-SLNs. Comparing ATR-SLN1 with ATR-SLN3 showed that, generally, an increase in the lipid concentration of SLNs leads to an increase in the particle size from 93.73 ± 5.65 to 143 ± 2.19 nm (*p* = 0.0023). The table also shows that the drug entrapment efficiency (EE %) varied from 38.72 ± 1.14 to 49.98 ± 0.9% (*p* = 0.0026). The data obtained in this study were in agreement with other studies reported (22,23).

### Effect of HLB of Surfactants on Nanoparticles Properties

**Fig. 1:**
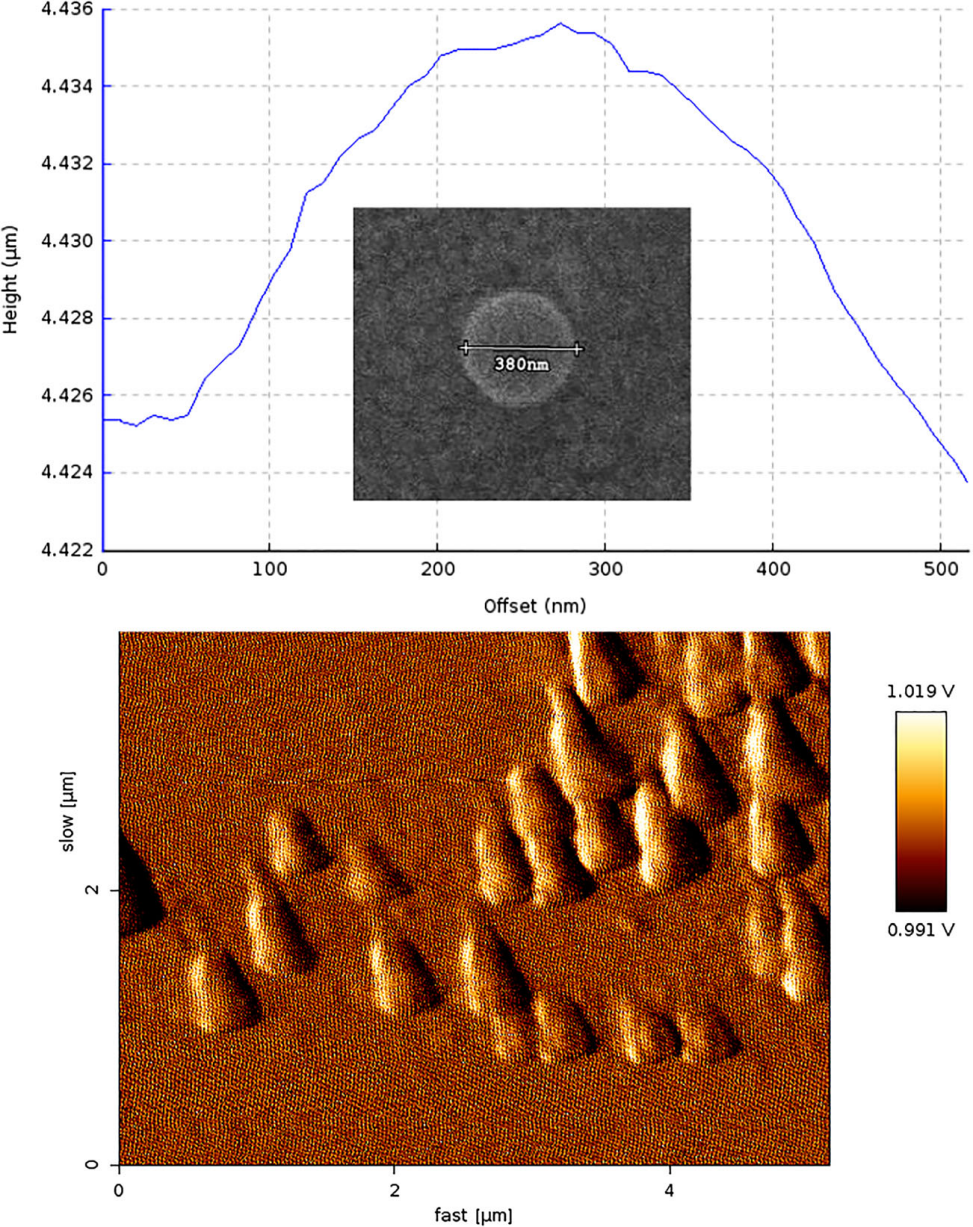
SEM and AFM micrographs of ATR-solid lipid nanoparticles (ATR-SLN7)

As the main goal was to overcome the dermal delivery issue of atorvastatin, therefore, atorvastatin solid lipid nanoparticle delivery *via* skin was optimized by changing the HLB values between 9.1 and 15.6 in the preparation of SLNs. The polydispersity index (PDI) of nanoparticles could be in the range of 0 to 1. Values above 0.7 are an indication of a very broad size distribution (24). PDI less than 0.3 is considered very homogenous populations of SLN. Based on Table I, the prepared SLNs had a PDI value of ≤ 0.5, which can be acceptable (25–30). The particle size distribution of the optimized formulation and some of the solid lipid nanoparticle formulations are shown in supplementary materials (Fig. 1S). The size distribution shown in Fig. 1S exhibited that in some cases, there was more than one peak which could be an indication of the presence of microparticles in the formulations.

Based on the properties which are listed in Table I, a decrease in the HLB value increases the size, EE%, and zeta potential (better stability) of nanoparticles, The smallest nanoparticles and PDI were obtained for ATR-SLN4 (71.07 ± 1.72 nm) which indicates the HLB value (15.6) was at the optimum level (generally, emulsion droplet can be the smallest at the optimum HLB value).

As the loading amount can be influenced by the solubility and miscibility of the drug in the lipid matrix, therefore, higher EE% due to a reduction in the HLB could be as a result of the high solubility of atorvastatin in Span 80 (solubility of atorvastatin calcium in span 80 is 47.47 mg/ml) (31). Reduction in the HLB value is accompanied by increasing the amount of Span 80 which can help to load atorvastatin (log *p* = 6.98 and water solubility is 0.00063 mg/ml) into the solid lipid (14). As the smaller particle size has a higher surface area, therefore, the drug molecules have more tendency to escape from the lipid matrix which resulted in lower EE% (8). The authors of this research article have also shown in other studies that an increase in the percentage of EE% could be attributed to the high solubility of naproxen in Span 80 (naproxen log *p* = 3.39 and water solubility is 0.0299 mg/ml) (14) or larger particle size in the case of metformin (log *p* = − 1.8 and water solubility is 1.38 mg/ml) (15). However, the increase in EE% due to an increase in the amount of Span and particle size was not the case for ATR-SLN8 where the amount of Span becomes more and the particle size becomes larger. This is an interesting result as an increase in EE% was expected due to an increase in the amount of Span and particle size. There may be an optimum level to increase the EE% or maybe the amount of Span and the size of particles are not the only two parameters which control EE%. It is believed that an increase in EE% could be due to the tendency of Span to form micelles which could dissolve atorvastatin in the solution or absorbed into the lipid matrix and increase EE%. This indicates that to reach desirable particle size and EE%, the ratio of surfactants (HLB value) used in the preparation of SLN should be optimized.

The results showed that the range of zeta potential for these formulations (containing non-ionic surfactants) was in the range of − 7.7 ± 0.61 mV (F4) to − 17 ± 0.87 mV (ATR-SLN8) (*p* = 0.0024). This negative charge around the particles may be due to the presence of residual electrolyte as a result of the ethoxylation catalyst of non-ionic surfactants (32), or maybe owing to the dipole nature of the ethoxy groups of Tween and Span (33). According to Table I, the zeta potential was increased with an enhancement in the amount of Span 80 in the formulation. It is generally expected that at the optimum level of a binary mixture of surfactants, a stable surfactant film at the interface of emulsion droplets is formed which could be leading to fine droplets with better stability (34). It seems that a more condensed barrier around particles could be formed with an increase in the Span concentration which makes particles more negatively charged (Table I). Generally, the zeta potential value is an indication of particle surface charge (the presence of lipid and surfactants) and the Stern layer (the presence of a free drug in the aqueous medium). When the zeta potential of metformin and naproxen solid lipid nanoparticles is compared with the zeta potential of the formulations obtained for atorvastatin, it can be concluded that nanoparticles are more negatively charged in the current study (− 7.7 ± 0.608 mV to − 17 ± 0.866 mV) than when metformin (− 0.651 ± 0.31 mV to − 6.180 ± 0.44 mV) (15) or naproxen (− 3.92 ± 0.71 mV to − 10.57 ± 0.57 mV) (14) was used. In the case of metformin, very low zeta potential could be due to the presence of a positively charged drug which makes zeta potential less negative.

A study performed by Elmowafy *et al.* (35), where surfactants have been used to prepare nanolipid carrier of atorvastatin by ultrasonication method, has shown that the particle size range has been between 162.5 ± 12 and 865.55 ± 28 nm for various formulations, and zeta potential values varied between − 34 ± 0.29 and − 23 ± 0.36 mV. They have also shown that EE% could increase to 96.6%. The particle size range produced in our study was narrower (71 to 202 nm for various formulations) than Elmowafy’s study, but their encapsulation efficiency was much higher compared with the current study (34.9–53.8%). In contrast to our study which SLN was designed for dermal delivery, their SLN has been suitable for oral delivery. Most of the studies carried out on atorvastatin solid lipid nanoparticles have been for oral delivery (35–38) rather than dermal or transdermal delivery. Depending on the composition of SLN varying particle size, zeta potential, PDI, and EE% can be obtained (35,38).

### Scanning Electron Microscopy and Atomic Force Microscopy

Particle size and morphology of nanoparticles were studied by SEM (SNE-4500M) and AFM (JPK-AFM). The images obtained for ATR-SLN7 (the highest EE%) by SEM and AFM (Fig. 1) showed that the particles were uniform in size and spherical based on the SEM image and were segregated based on the AFM image.

### FTIR Spectroscopic Analysis

**Fig. 2:**
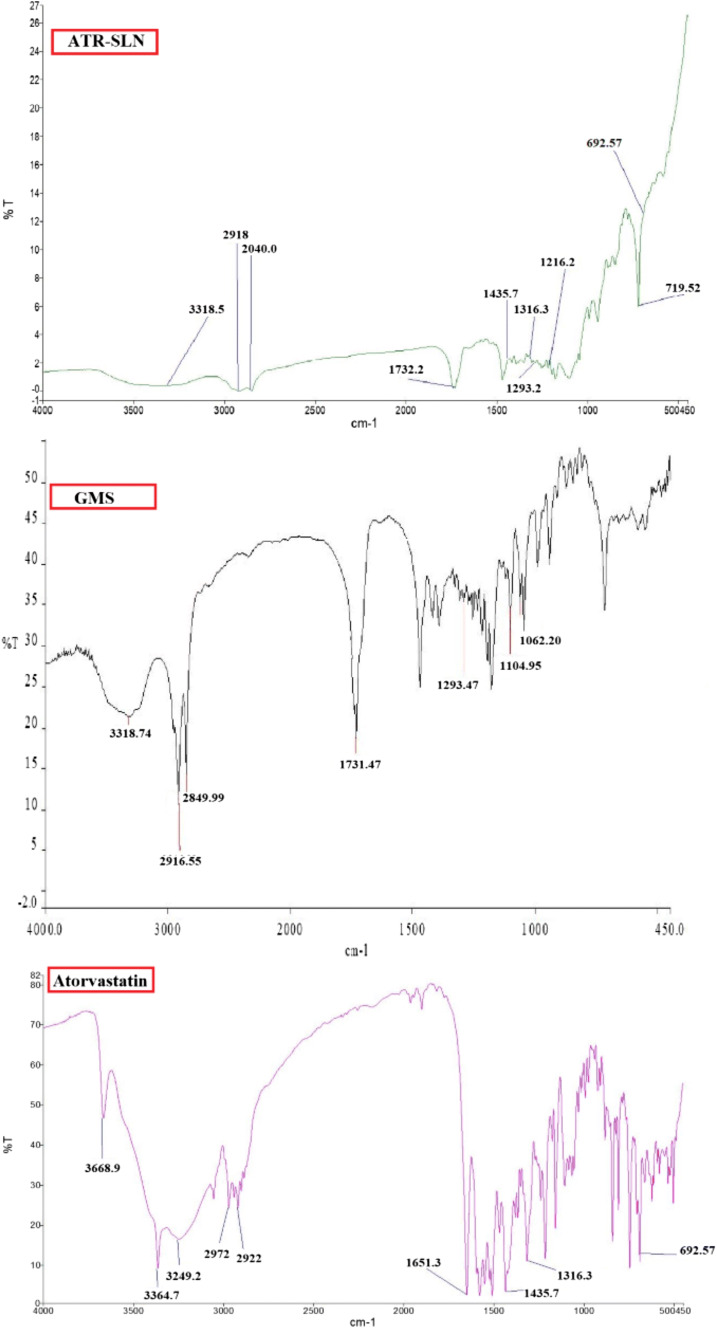
FT-IR spectra of ATR (atorvastatin), GMS (glyceryl monostearate), and the optimized ATR-SLN7 (atorvastatin-solid lipid nanoparticles)

To explore any changes in the FTIR of the chemicals used in the preparation of atorvastatin SLN (the optimized formulation 7), the infrared spectra of ATR, GMS, and ATR-SLN7 were obtained and shown in Fig. 2. Atorvastatin (ATR) showed characteristic bands at 3668 cm^−1^ and 3249.2 cm^−1^ (O–H stretching), 3364 cm^−1^ (N–H stretching), 1651 cm^−1^ (C=O of amide stretching), 1316 cm^−1^ (C–N stretching), and 692 cm^−1^ due to aromatic out-of-plane bend. GMS showed a broad peak at 3400–3200 cm^−1^ which corresponds to O–H stretching. There were also important peaks in the FTIR spectrum of GMS at 3000–2850 cm^−1^ (C–H stretching), 1731 cm^−1^ (C=O stretching), and 1300–1000 cm^−1^ (C=O stretching). FTIR results ruled out any chemical interaction between the drug and other components of the SLN.

### Differential Scanning Calorimetry

**Fig. 3:**
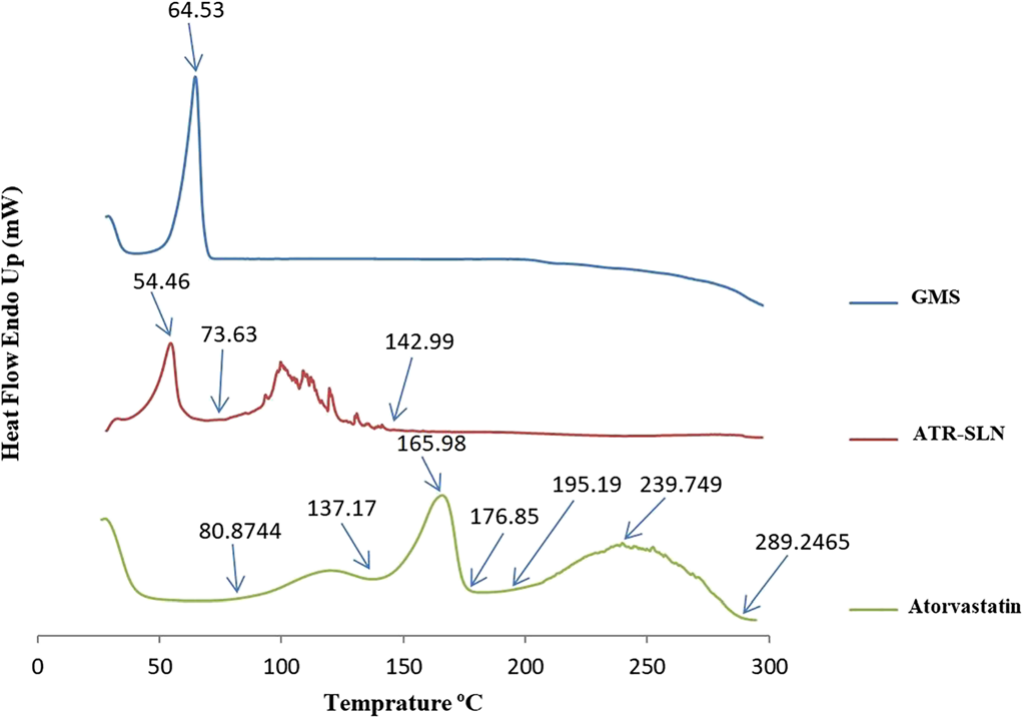
DSC traces of ATR (atorvastatin), GMS (glyceryl monostearate), and the optimized ATR-SLN7 (atorvastatin-solid lipid nanoparticles) scanned at a heating rate of 10°C/min

DSC was performed for the optimized formulation (ATR-SLN7), GMS, and plain atorvastatin, and the results are shown in Fig. 3. The DSC traces for GMS exhibited an endothermic sharp peak at 64.53°C corresponding to its melting point. ATR DSC traces present 3 steps in the range of 80.87 to 137.17°C, 137.17 to 176.85°C, and 195.19 to 289.24°C respectively. The steps are associated with loss of water, melting point, and degradation (phase transition) of atorvastatin respectively. Atorvastatin melting peak was observed at 165.98°C. Comparing DSC traces of GMS and atorvastatin with the DSC traces of ATR-SLN7 show a single endothermic peak around the GMS melting point, and the endothermic peak for melting of ATR is missing. This indicates that ATR in SLN formulations could be in a molecular state or if it is in a crystalline state due to a very low concentration of ATR in the formulation, it is not detectable. Furthermore, the absence of a diagnostic peak of ATR in the formulation could be a result of the dissolution or miscibility of the atorvastatin in the molten GMS. The formulation showed the endothermic peaks at a slightly lower temperature (for example GMS has an endothermic peak at 64.53°C which can be seen at 54.46 in formulation). These differences and also decline in melting point could be attributed to the nanometric size of particles as a result of a high specific area (large surface to volume ratio) (9,15,39).

Furthermore, the shift in GMS peak could be due to the presence of surfactants (Tween 80 and Span 60) in SLN formulations which facilitates the miscibility of GMS with the surfactants in SLN. Similar results have been reported by Trivino *et al.*, (40) when the miscibility of drug-lipid-surfactant has been investigated *via* DSC. They have reported that a reduction in the melting point of GMS in the mixtures with surfactants could be due to the miscibility of GMS in surfactants. In another study, it has been reported that the decrease in the melting temperature of GMS in SLN formulation could be explained as a result of the conversion of GMS into stable β form during heating and cooling operations in SLN preparation (41) which also has been discussed in details elsewhere (42).

**Fig. 4:**
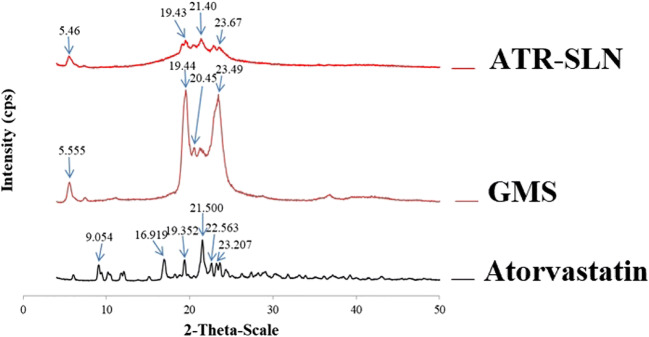
XRD of atorvastatin, GMS (glyceryl monostearate), and the optimized ATR-SLN7 (atorvastatin-solid lipid nanoparticles)

Figure 4 also shows that the transition phase or degradation event disappeared in SLN formulation. The author believed that this probably could be due to the dissolution or miscibility of ATR in the molten lipid/surfactant during DSC run which could protect atorvastatin molecules from degradation. This protection does not exist when pure atorvastatin was used due to the absence of lipid/surfactants. The dissolution or miscibility of ATR in the lipid or surfactants could explain the disappearance of the ATR melting peak in SLN formulation. A similar conclusion has been reported when the thermal behavior of binary mixtures of ATR-mannitol or ATR-lactose is studied (43).

### Powder X-ray Diffractometer Analysis

X-ray diffraction (PXRD) patterns of ATR, GMS, and ATR-SLN (ATR-SLN7) are depicted in Fig. 4. ATR revealed several diagnostic peaks at 2θ: 9.054°, 16.919°, 19.352°, 21.500°, 22.563°, 23.207°, and 23.596°. The GMS shows the peaks at 2θ: 5.555°, 19.44°, 20.45°, and 23.49°. These sharp peaks for ATR and GMS are an indication of the highly crystalline nature of these two chemicals. The PXRD pattern of SLN of atorvastatin clearly shows the diagnostic peaks of ATR but a reduction in the intensities of the peaks due to the low concentration of ATR in the SLN formulation. The peaks at 2θ: 5.46°, 19.43° may be attributed to GMS and peaks at 21.40°, 23.67° could be attributed to ATR (44). The results are aligned with FTIR and DSC analysis which showed good incorporation of ATR in the nanoparticles without any chemical interaction.

### *In Vitro* Percutaneous Absorption Study

The World Health Organization (WHO) has the guidelines for membranes obtained from various sources that can be employed for *in vitro* skin permeation/penetration studies (45). Among all models that have been developed for evaluation of *in vitro* skin permeation and penetration, rat skin is one of the most popular models which has been widely used. Although rat skin is more permeable than human skin, still, it is acceptable to choose rat skin instead of human skin in the early phases of development because of practicality and economical reasons (46).

**Fig. 5:**
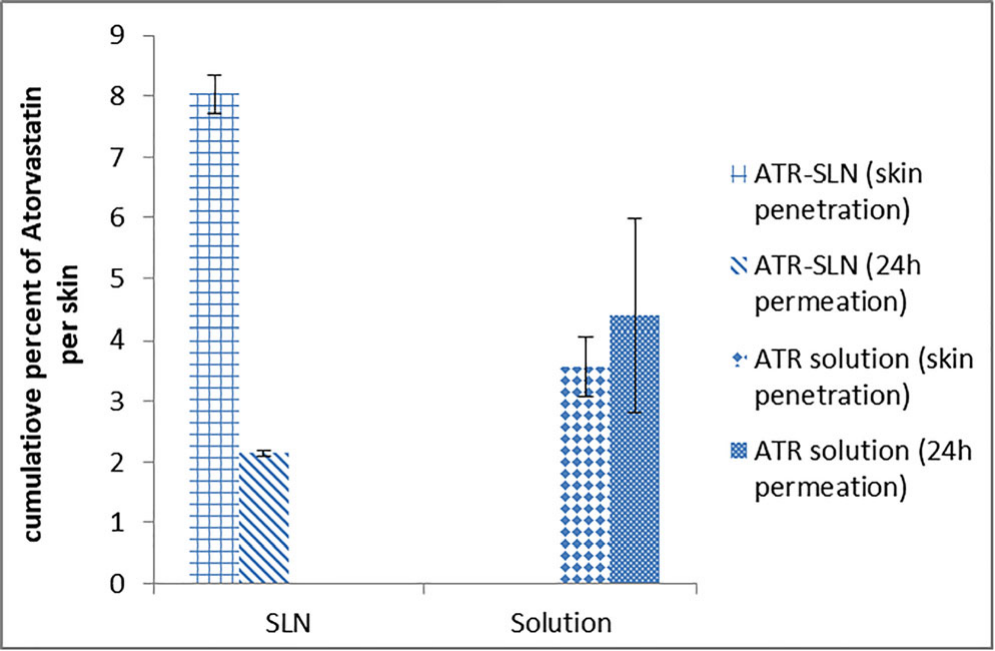
Amount of ATR penetrating to the skin layers (dermal delivery) and permeated (transdermal delivery) (error bars are standard deviation; the sample represents ATR-SLN and standard formulation is the ATR solution containing the same concentration of ATR; *n* = 3)

**Fig. 6:**
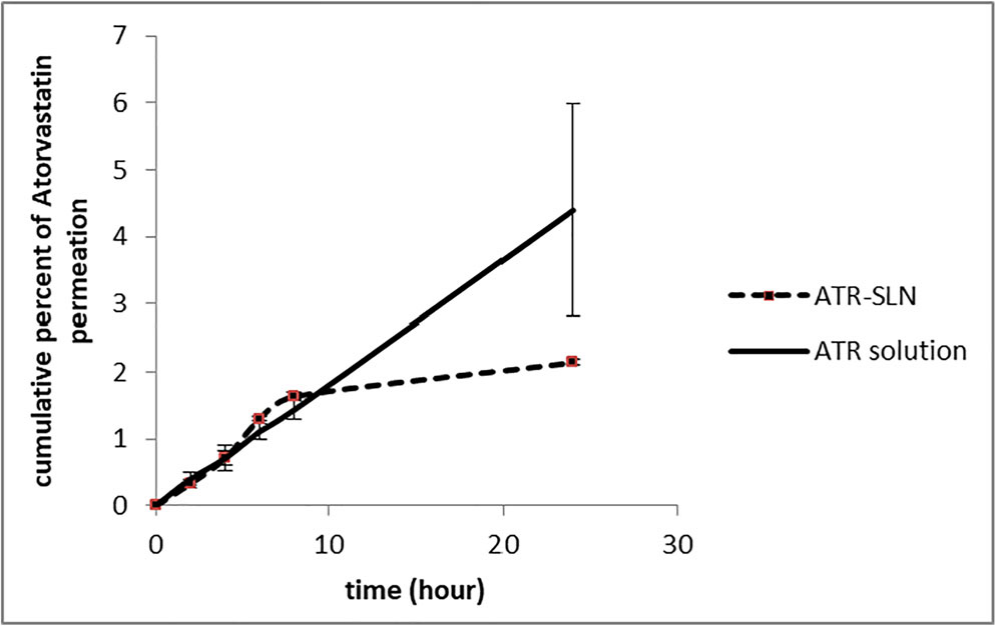
Cumulative percentage of ATR (atorvastatin) permeated across rat skin (data is the mean and standard deviation of three determinations, *n* = 3; the sample represents ATR-SLN7 and standard is ATR solution containing the same concentration of ATR exist in nanoparticles

In this study, to evaluate the skin permeation and skin absorption properties of SLN formulation, ATR-SLN7 was selected as the optimum formulation and its counterpart formulation (atorvastatin ethanolic solution with a similar concentration as SLN7, Tween and Span, except lipid) was used as the standard. Figure 5 shows the cumulative percentage of plots of atorvastatin penetrated (dermal delivery) and permeated (transdermal delivery) for SLN and solution. Although the amount of ATR permeated through the skin was higher for the standard solution compared with SLN formulation, the increase in permeation of the drug was not significant (*p* > 0.0697) between these two formulations (Fig. 5), whereas the amount of ATR penetrated to skin layers was higher for SLN (ATR-SLN7) (*p* < 0.0002) (Fig. 5). Figure 6 shows the cumulative plots of permeated (transdermal delivery) amount of atorvastatin through the skin from atorvastatin alcoholic solution and SLN. It is obvious from this figure that atorvastatin can permeate the skin at high concentration than SLN formulation after 10 h. This indicates that SLN formulation is suitable for dermal delivery compared with ATR solution as it stays more in the skin layers. On the other hand, ATR solution is more suitable for transdermal delivery compared with SLN formulation as more drugs can penetrate through the skin when the solution was employed. The results highlight the property of SLNs as a suitable carrier for efficient dermal delivery for atorvastatin with less systemic absorption. Furthermore, a prolonged anti-inflammatory effect with low adverse effects could be expected (4,47,48) for ATR-SLNs as the results showed that solid lipid nanoparticles can improve the accumulation into the skin opposed to its ethanolic solution which showed less accumulation of the drug in the skin layers (49). The results showed that the solid lipid nanoparticles reduce the drug permeation and also increase accumulation in the horny layer; they also showed SLNs could prolong the anti-inflammatory effect. To calculate the amount of drug retained in the stratum corneum (non-viable layers) in the current study, extra experiments such as stripping are needed. As in the current study, dermal delivery involved both layers (viable and non-viable layers); therefore, this experiment was not carried out. In addition, it is obvious from Fig. 6, any formulation that moves slower in the skin layers can increase the chance of the drug remaining in the skin layers (dermal delivery) which is the case for atorvastatin SLN.

In order to achieve a better anti-inflammatory effect of ATR, the drug should reach viable skin layers (epidermis and dermis) with high concentration. Our results show that SLN containing ATR can prolong the drug release; therefore, this can potentially increase the length of stay of drug molecules in the viable skin layers to induce the anti-inflammatory effect. Accordingly, it can also be stipulated that higher concentrations of ATR formulation may increase the drug concentration in these layers even further. These will need to be tested in future investigations using the exact determination of the drug in the dermis and epidermis.

Overall, the results showed that, although the total amount of dermal and transdermal delivery for both formulations was around 10%, the contribution of each formulation regarding dermal and transdermal delivery is different which was the main aim of the current study. This is important as if the transdermal delivery is needed then the ethanolic solution could be better than SLN, whereas, for dermal delivery, SLN formulation is better than the ethanolic solution of atorvastatin. It should be noted that this is true for atorvastatin and it could be different if the type of drug changes.

## CONCLUSION

The results highlight the property of SLNs as a suitable carrier to carry atorvastatin to the site of disease. On the other hand, SLNs could decrease systemic absorption and keep the drug longer at the site of action. Therefore, a prolonged anti-inflammatory effect with low adverse effects could be expected from atorvastatin solid lipid nanoparticles. The results showed that the SLN containing atorvastatin can be used as an efficient and promising formulation to localize atorvastatin in skin layers as an anti-inflammatory agent for the treatment of scalp seborrheic dermatitis. To achieve the mentioned targeted delivery, a stable formulation with minimum batch to batch variation with a suitable dermal delivery property (smaller particle size, smaller PID, and higher EE %), the optimization of SLN formulations by changing the contribution of binary mixtures of non-ionic surfactants is necessary.
